# Processes affecting altitudinal distribution of invasive *Ageratina adenophora* in western Himalaya: The role of local adaptation and the importance of different life-cycle stages

**DOI:** 10.1371/journal.pone.0187708

**Published:** 2017-11-10

**Authors:** Arunava Datta, Ingolf Kühn, Mustaqeem Ahmad, Stefan Michalski, Harald Auge

**Affiliations:** 1 Department of Community Ecology, Helmholtz Centre for Environmental Research–UFZ, Theodor-Lieser-Straβe 4, Halle, Germany; 2 Institute of Biology/Geobotany and Botanical Garden, Martin-Luther-University Halle-Wittenberg, Am Kirchtor 1, Halle, Germany; 3 German Centre for Integrative Biodiversity Research (iDiv) Halle-Jena-Leipzig, Deutscher Platz 5e, Leipzig, Germany; 4 High Altitude Biology, CSIR—Institute of Himalayan Bioresource Technology, Palampur-, Himachal Pradesh, India; INRA - University of Bordeaux, FRANCE

## Abstract

The spread of invasive plants along elevational gradients is considered a threat to fragile mountain ecosystems, but it can also provide the opportunity to better understand some of the basic processes driving the success of invasive species. *Ageratina adenophora* (Asteraceae) is an invasive plant of global importance and has a broad distribution along elevational gradients in the Western Himalayas. Our study aimed at understanding the role of evolutionary processes (e.g. local adaptation and clinal differentiation) and different life history stages in shaping the distribution pattern of the invasive plant along an elevational gradient in the Western Himalaya. We carried out extensive distributional surveys, established a reciprocal transplant experiment with common gardens at three elevational levels, and measured a suite of traits related to germination, growth, reproduction and phenology. Our results showed a lack of local adaptation, and we did not find any evidence for clinal differentiation in any measured trait except a rather weak signal for plant height. We found that seed germination was the crucial life-cycle transition in determining the lower range limit while winter mortality of plants shaped the upper range limit in our study area, thus explaining the hump shaped distribution pattern. Differences in trait values between gardens for most traits indicated a high degree of phenotypic plasticity. Possible causes such as apomixis, seed dispersal among sites, and pre-adaptation might have confounded evolutionary processes to act upon. Our results suggest that the success and spread of *Ageratina adenophora* are dependent on different life history stages at different elevations that are controlled by abiotic conditions.

## Introduction

In the process of colonizing new geographic areas, invasive plant species often spread along environmental gradients and become successful in diverse environmental conditions [1]. The spread of invasive plants along such environmental gradients offers the opportunity to study the mechanisms underlying successful biological invasions [2]. Exceptionally steep environmental gradients in mountains across relatively short geographic distances provide a quasi-experimental setup to investigate the fundamental processes that drive the spread of invasive plants. Although mountains have often been considered relatively resistant to plant invasions, recent studies show that invasive plant species have also colonized higher elevations across the globe [3–6]. Invasions in mountain areas are of practical concern since many mountain ecosystems are biodiversity hotspots and source of important ecosystem services [7,8]. In addition, invasive plants are difficult to manage in the mountains because of inaccessible and rugged terrain and hence might become uncontrollable after successful naturalization [3].

One mechanism behind the successful spread of some invasive plants across a broad elevational range is rapid adaptive evolution. Multiple exotic plant species have undergone adaptive genetic divergence along elevational gradients [9] and established elevational clines similar to native species [10]. Environments at the elevational range margins may impose strong selection pressure, leading to adaptive divergence of populations at upper as well as lower range margins [11,12].

It has been suggested that rapid evolutionary processes such as local adaptation might play a significant role in the spread of invasive plants in the naturalized range [13,14]. If there is a strong selection pressure, sufficient genetic diversity and isolation of populations [15,16], adaptive evolution can occur on very short temporal scales [17,18]. Adaptive divergence among plant populations is a rather common phenomenon [19] and occurs as frequently among invasive plant species as among native plants [20]. For instance, it has been repeatedly shown that invasive plant species may rapidly build up latitudinal clines [21–23]. Founding populations are often genetically impoverished due to the small population size and hence suffer from genetic bottlenecks [24]. Accordingly, single introduction events may cause severe genetic bottlenecks in invasive species which may hamper local adaptation [25]. Although rapid evolutionary changes during invasions have been frequently inferred in spite of genetic bottlenecks, they might be non-adaptive as revealed by simulations models [26]. Apart from local adaptation, the ability of a genotype to exhibit different phenotypes under different environmental conditions (i.e. phenotypic plasticity) has been considered to play a crucial role in plant invasion as phenotypic plasticity allows naturalization along a broad range of environmental conditions [27,28]. In situations when the genetic make-up prevents adaptive evolution, invasion success across environmental gradients may be facilitated by pre-existing phenotypic plasticity [14].

Although plant demography can be considered crucial to understanding range dynamics [29], knowledge about demographic processes at range margins is still insufficient [30]. In general, harsh environments at upper and lower elevational range margins translate into strongly reduced plant fitness, with range-edge populations often acting as demographic sinks [31,32]. Most of the exotic species studied so far along elevational gradients show a continuous decrease in frequency of occurrence with increasing elevation ([32,33]; but see [34] for exceptions). This pattern is due to unidirectional spread from the lowlands to higher elevations, associated with environmental filtering because only a few invaders succeed under the extreme environmental conditions at high elevations [33]. In contrast, many native plant species in montane environments attain maximum frequency at mid-elevation, resulting in a unimodal distribution along elevational gradients [35]. Interestingly, such a pattern has been less often documented for exotic species so far but may be expected if abiotic or biotic conditions are sub-optimal both at low and high elevations.

The type of breeding system may have consequences for invasion success because it influences the genetic structure of the invasive population. Invasive plants that reproduce sexually are able to maintain higher genetic diversity due to recombination compared to species that reproduce clonally. On the other hand, clonal plants have the advantage of reproducing independently without any pollen limitation and are able to maintain trait expressions that confer invasiveness. Interestingly, several plant species that reproduce clonally have been very successful invasive plants as well. For example, several members of Asteraceae that reproduce apomictically are known to be aggressive invaders (e.g. *Ageratina adenophora*, *Eupatorium adenophorum*, *Ageratina riparia*) across tropical and sub-tropical regions of the world [36,37]. Despite the fact that adaptive evolution in these apomictic plants is limited due to genetic constraints, they are able to colonize huge geographical areas and hence are interesting target species to understand eco-evolutionary processes leading to their success.

To better understand the processes leading to successful invasion, we studied invasive populations of the apomictic plant species *Ageratina adenophora* in western Himalaya. This species is a perennial plant native to Mexico and is invasive in subtropical regions worldwide. We choose this species since it is one of the few invasive plants having a broad elevational distribution. Being a subtropical species, the lower range limit along an elevational gradient might be imposed by hotter and drier climatic conditions while the upper range limit might be determined by low temperature. Reciprocal transplant experiments carried out in China, where *A*. *adenophora* was introduced ca. 40 years ago, revealed no evidence for local adaptation along an elevational gradient [38,39]. In the Himalayas, however, studies on evolutionary processes behind its successful spread across a broad elevational range are lacking.

Reciprocal transplant experiments enable us to study local adaptation [13,40] by rigorously testing whether resident genotypes perform better than those introduced from other sites (‘local vs. foreign’ criterion: [41]). Additionally, regressing traits measured in a common garden against environmental conditions [42,43] or geographic coordinates of home sites of populations [2,21,23] allows us to find evidence for clinal differentiation. We combined both approaches, by transplanting offspring of local *A*. *adenophora* populations from each of three elevational levels (at 570 m, 1330 m and 2100 m a.s.l. on average) into three common gardens, one at each elevation. We studied how germination, growth, survival and reproduction are limited by the particular environments, whether populations perform best at or close to their “home” elevation, and whether there is evidence for clinal variation along the elevational gradient. Combined with extensive field surveys in the western Himalaya we used these experiments to answer the following questions:

1. Is the distribution of invasive *A*.*adenophora* in the western Himalayas limited by environmental conditions at both, low elevations and high elevations, leading to a unimodal pattern of occurrence?
2. Which life-cycle stages are most vulnerable at the elevational range margins of this species?
3. Is there any evidence of rapid evolutionary changes in the western Himalayan populations of *Ageratina adenophora* in form of local adaptation and clinal differentiation?

## Methods

### Study species

*Ageratina adenophora* (Spreng.) King & H.Rob. is a herbaceous, perennial, triploid Asteraceae native to Mexico. It has naturalized in more than 30 countries across the globe and is considered to be a noxious invasive plant in south Asia, east Asia, south east Asia, eastern coast of Australia, and South Africa [44–46]. Invasive attributes of *A*. *adenophora* include high reproductive rate due to uniparental reproduction by apomixes [36,47,48] and vegetative propagation [49], strong allelopathic effect [50,51], and effective wind dispersal of the seeds [49,52]. The plant shows luxuriant growth in cool moist regions along the slopes of hills or mountains but it is capable of growing in diverse conditions. The plants invade new regions along road verges and rivers which form a conduit for its dispersal (for more details about the plant, see S1 Appendix) [53].

### Distribution survey

The distribution survey was carried out in a region of western Himalaya between 29.96° and 32.55°N and 75.77° and 78.43°E, and elevations between 300 m and 4100 m (for details of survey refer to S2 Appendix) in 2015 and 2016. Previous reconnaissance surveys and existing literature on the distribution of *Ageratina adenophora* in the Himalayas [54,55] had indicated that the plant has naturalized in the elevational band between 300 m to 2500 m a.sl (in subtropical and sub-temperate zones) and is completely absent at high elevations beyond 3000 m. We, therefore, conducted our survey primarily in the elevational zone between 300 m and 2500 m but surveyed also some areas beyond the known elevational range of the plant.

The distribution survey was carried out in haphazardly chosen locations between 300 m to 3000 m elevational belt representing diverse landscapes (such as forest land, urban and suburban areas, rural areas, agricultural fields, riversides, flood plains and dams etc.). Although most of the survey was carried out along road sides, high elevational areas (beyond 2500 m) were surveyed using the trekking routes. A total of 389 locations were surveyed as the presence-absence status was recorded.

### Common garden experiments

#### Seed sampling

**Fig 1:**
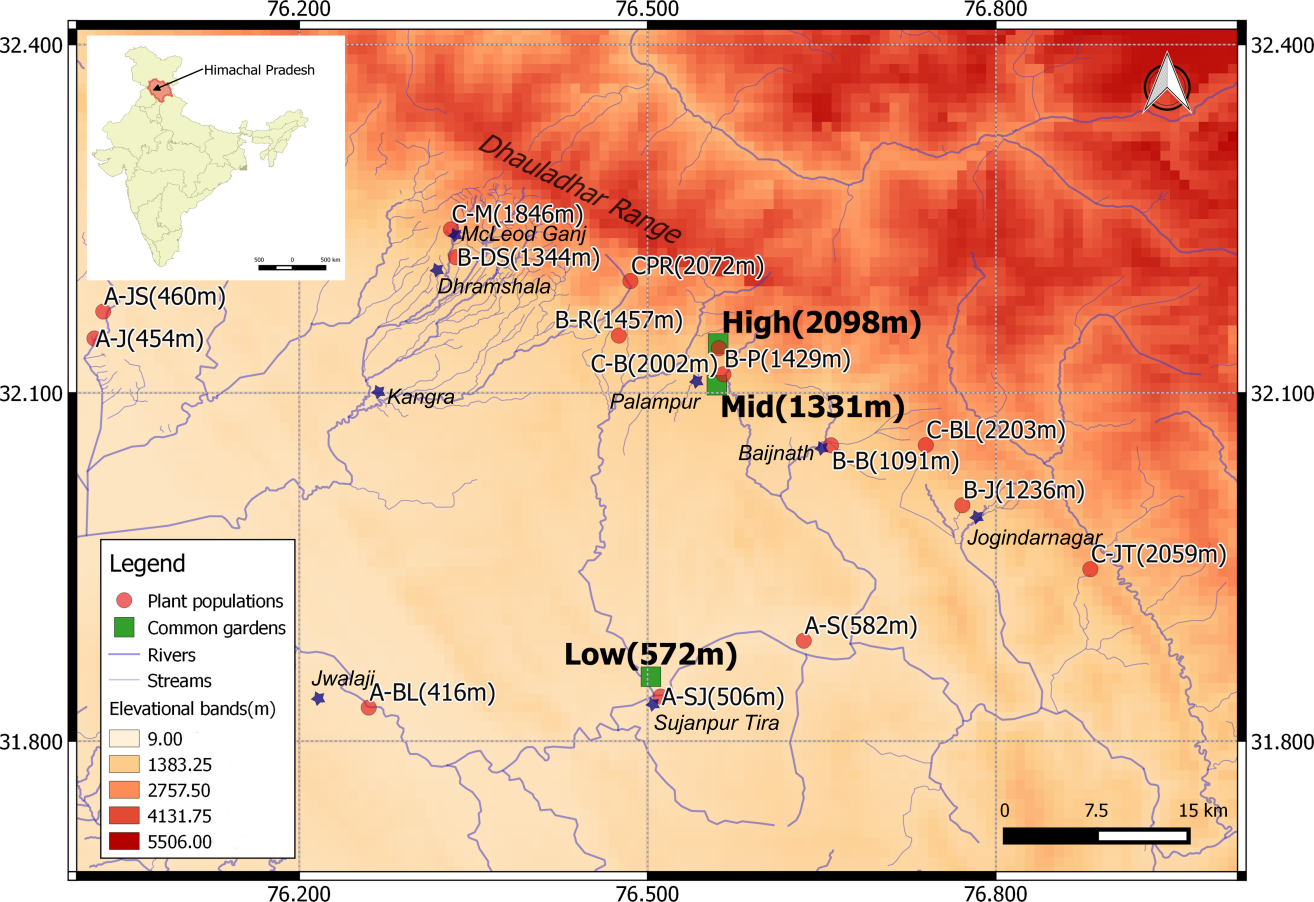
Locations of the 15 *Ageratina adenophora* populations and of the three common gardens that are located on the southern aspect of Dhauladhar mountain range in Himachal Pradesh (India). The populations and common gardens are denoted by red circles and green squares respectively. Important places in the vicinity are indicated by blue stars.

Based on the distributional survey, we divided the elevational range of *A*. *adenophora* into three elevational belts in the southern aspect of Dhauladhar range (Kangra District, Himachal Pradesh, India; see Fig 1), i.e. low (400 m-600 m), mid (1100 m-1500 m) and high (1800 m-2200 m) elevations, termed “origins” hereafter (for site-specific climatic conditions see S2 Table). Within each of the three belts, we randomly selected 5 populations, well separated in space and with a minimum population size of ten individuals. Within each population, we collected seeds from five randomly chosen individuals, termed as “seed families” hereafter. Our hierarchical sampling design finally resulted in seeds from 75 seed families representing 15 populations and three elevational origins. Since the plants at low elevational garden flowered earliest, we began sampling at the lowest origin (1^st^ week of April 2014), followed by the mid origin (4^th^ week of April 2014) and finally the highest origin (2^nd^ and 3^rd^ week of May 2014). Floral heads of each maternal plant were stored separately in paper bags and air dried at room temperature. After drying, seeds were separated from other floral parts and stored in vials with dehydrated silica gel at room temperature.

#### Experimental design and measurements

In May-June, 2014, we prepared one common garden at each of the three altitudinal levels in the Dhauladhar range: a low- elevation garden at 570 m, a mid-altitudinal garden at 1330 m, and a high-altitudinal garden at 2100 m. In addition to climatic conditions (S2 Table), the common garden sites differed significantly in soil chemical parameters (see S4 Table). Seedlings were grown in nurseries (approximately 4.8 m×1.8 m) adjacent to each common garden. The nurseries were covered with green shading net (which allowed about 50% light transmission) to prevent excessive evaporation. Seeds were germinated in small polythene bags (volume approximately 180 cm^3^) filled with a mixture of equal proportions of clay, soil and farm yard manure. Groups of ten polythene bags were assigned to each of the 75 seed families and randomly positioned in the nurseries. Seeds were sown in the 1^st^ week of July 2014 and were watered regularly in order to maintain adequate soil moisture (see S3 Table for exact dates). After the seeds germinated, 2–3 seedlings were kept in each polythene bag and any extra seedling was removed at an early stage. The seedlings were maintained for approximately six weeks after sowing (until 3^rd^ - 4^th^ pair of leaves appeared) before transplanting them to the common garden.

Each of the common gardens had a size of 6.4 m × 27 m and was divided into five blocks, arranged along the slope of the gardens. One randomly chosen individual from each of the 75 seed families was randomly assigned to a planting position within each of the blocks (arranged in 25 rows and 3 columns). Distance among seedlings was approximately 0.22 m. This design resulted in 375 (75×5) individual plants arranged randomly in five blocks in each of the three common gardens, and a total of 1125 individual plants (375×3) in the entire experiment. Seedlings were transferred along with their root ball including the potting mixture into small holes made in the soil and were watered regularly for the next two weeks. Seedlings that died within one week after transplantation were replaced with new seedlings from the nursery (see S3 Table for exact dates of seed sowing and transplantation). No specific permissions were required to conduct our study at any of the locations and the study did not involve any protected or endangered species.

During the course of the experiment, we measured various plant functional traits: The onset of flowering was recorded daily from 8^th^ March 2015 until all the plants flowered. A plant was considered to be flowering when at least one floret in the capitulum had opened. The number of days to flower for a plant was calculated as Julian days (i.e. days from 1^st^ of January 2015). To measure specific leaf area (SLA) and leaf dry matter content (LDMC), we sampled five mature and non-senescing leaves from the upper branches of the plant in July 2015 and stored them immediately between moist tissue papers inside a zip-lock bag. Leaves were kept cool in an ice box while transporting them to the laboratory and then stored in a refrigerator at 4°C. Fresh weight was measured within 24–48 hours and dry weight was measured after drying the leaves in an oven at 60°C until constant weight was achieved. Leaf area was determined by scanning the leaves along with a scale in a flatbed scanner at 300 dpi and then analysing the images using ImageJ software (by converting the images to grayscale and then applying the default thresholding function to delineate the background). SLA was calculated by dividing the one-sided area of the fresh leaf (in cm^2^) by the oven-dried biomass (in mg). LDMC obtained by dividing oven leaf dried weight of the leaf (in mg) by water saturated fresh weight of the leaf (in g). The plants were harvested in the 2^nd^ week of August 2015 (S3 Table). The plants were harvested at a height of approximately two centimetres above ground and the primary branches were counted from the base of the plant. Plant height and fresh weight were measured immediately after the harvest. Biomass (dry weight) could only be measured for a subsample of 150 plants per garden (due to logistic limitations) that were sampled across all blocks and populations. To obtain conversion factors to estimate the biomass of the remaining plants, we first performed an ANCOVA on the biomass of the 150 plants, with garden, block and population as factors and fresh weight as a covariate. Since block and garden had a significant effect on the relationship between biomass and fresh weight, we calculated the conversion factor specifically for each block in each garden. We then used the block-specific conversion factor to estimate the biomass of all remaining plants. Reproductive output of each plant was estimated by counting the number of capitula produced by each plant in April and May 2015. Since the plant reproduces apomictically [47], pollen limitation does not limit the production of viable seeds and hence count of capitula is a reasonable estimate of reproductive fitness of the plant.

#### Germination experiment

In addition to the main experiment, we conducted a germination experiment adjacent to the each of the three common gardens from 27^th^ July to 3^rd^ August 2014 in order to compare seed germination among origins, populations and seed families under nearly natural conditions. The germination experiment was performed in paper cups with perforated walls to allow the passage of soil moisture. Each cup was filled with autoclaved potting mixture (see above). The perforated sides of the cups were embedded in the soil to ensure moisture absorption and covered with a transparent nylon net protect to seeds from granivores. The experiment was laid in a randomized block design with three blocks. Each block had 75 paper cups, randomly assigned to the 75 seed families. Twenty seeds from a given seed family were sown in each paper cup. The number of germinated seeds in each cup was counted after one month and the experiment was terminated thereafter (see S3 Table for the experimental dates).

Since they are influenced by maternal provisioning [56,57], seed mass or initial seedling size can be considered as a proxy for assessing the influence of maternal effect (see for instance [40,58]). Therefore we included seed mass as a covariate when analysing germination probability, but we found no significant effect of seed mass.

#### Statistical analysis

In order to evaluate the distribution pattern of *A*. *adenophora* along the elevational gradient using presence-absence data collected along an elevational gradient, we used a set of seven hierarchical logistic regression models. These models, initially proposed by [59], were later implemented as “eHOF” package in R programming environment [60] by [61]. This set of models allows hypothesis testing and is hence considered to be more appropriate than alternatives offered by generalized additive models [61]. The first model in the hierarchy is a null model without any trend. The second model is a logistic response curve with increasing or decreasing trend. The third model is similar to the second model but the maximum is always below the upper limit of the data. The fourth model corresponds to a unimodal and symmetrical hump shaped response while a skewed unimodal response is modelled by the fifth model. The sixth and seventh models are designed for a bimodal response [61]. Amongst all the seven models, the best fitting model was judged based on the lowest AICc value (S5 Table). The elevation at which the probability of distribution of *A*. *adenophora* peaks along our elevational gradient was calculated as the maximum of the best fitting function.

Data from the common garden and germination experiments were analysed using generalized linear mixed models (SAS 9.4, proc GLIMMIX) with origin, garden and their interaction as fixed effects models, and population within origin, seed family within population and block within garden as well as population × garden and seed family × garden interactions as random effects. Biomass, the number of primary branches, SLA were analysed using a log-normal error distribution. Leaf dry matter content was logit transformed because it represents a proportion. The number of capitula and days to flower were square root transformed to approach normal distribution of residuals, while plant height and leaf area did not require any transformation. For analysing germination data we applied a model with binomial error distribution and logit link function. To account for possible maternal effects manifested in seed mass, we repeated the analysis with seed weight as a covariate. In all these models, we were particularly interested in the origin × garden interaction to test for local adaptation of populations considering the ‘local versus foreign’ criterion [41].To test for clinal differentiation of populations along the elevational gradient we applied an ANCOVA model on the population mean traits with elevation, garden and their interaction term as fixed effects.

## Results

### Elevational distribution pattern

**Fig 2:**
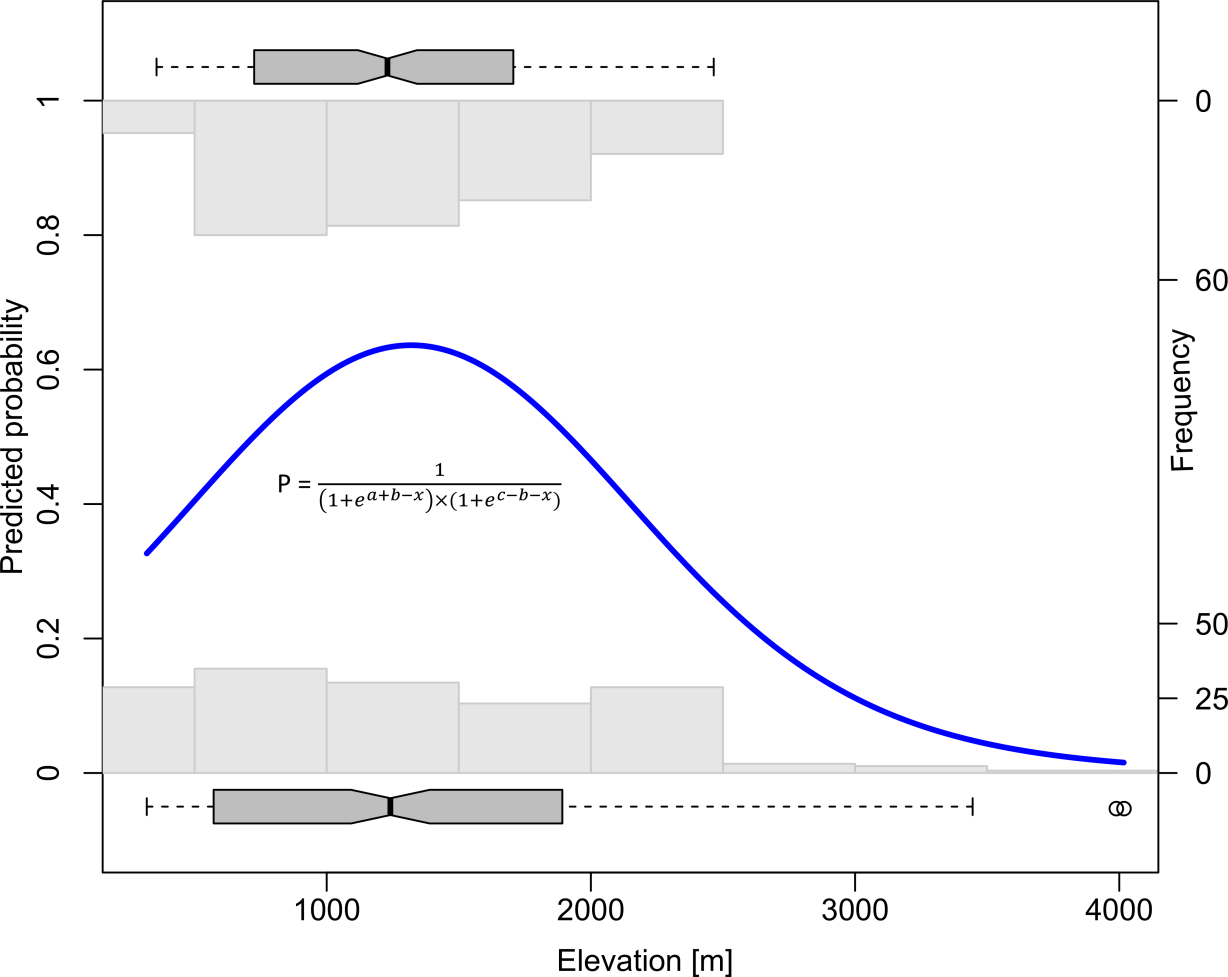
The probability of occurrence of invasive *Ageratina adenophora* along an elevational gradient in Western Himalaya shows a unimodal relationship with elevation. The bold blue line represents the predicted probability of occurrence from the model having best fit (according to the AICc values) out of the seven hierarchical models used [61]. The equation in the figure shows the function for calculating the probability of occurrence where *x* is the elevation (predictor) and *a*, *b*, *c* are parameters of the model that are estimated by maximum likelihood procedure. The marginal histograms on the top and bottom of the plot show the frequency of presences and absences respectively, while the marginal boxplots depict the spread of presences and absences along the elevational gradient.

*Ageratina adenophora* was present in 49.5% of the surveyed locations (193 out of 389 locations). Hierarchical regression analysis revealed that the distributional pattern of *Ageratina adenophora* along the elevational gradient was best explained by a unimodal and symmetrical model (Model IV, see S5 Table for details of the models). Elevational band between 1000 m to 1600 m had a high probability of occurrence and the peak probability of 0.63 was predicted at the elevation of 1319 m (Fig 2). The probability of occurrence at the lowest sampled elevation (319 m) was 0.32 while the probability of occurrence above 3000 m was less than 0.1 (Fig 2).

### Common garden experiments

#### Germination

In the open germination experiment conducted at the three elevations, all the seeds failed to germinate in the lowermost garden while 10.6 ± 3.4% and 9.8 ± 3.2% of the seeds (least square means ± standard error of the model) germinated respectively in the mid-elevation and high-elevation gardens. The lowermost garden was removed from statistical analysis since germination failed completely. Furthermore, seed weight did not affect the probability of germination (p = 0.196), hence we present only results of the model without seed weight as a covariate (Table 1). Analysis of germination data from the mid-elevation and the high-elevation gardens neither revealed a significant difference between the two gardens nor did the origin and the garden × origin interaction affect germination rate (Table 1).

**Table 1:** Results of mixed effect models comparing germination, growth- and fitness-related traits as well as leaf functional traits of invasive *Ageratina adenophora* populations between plant origins (low elevation, mid elevation, high elevation) and between gardens (mid elevation, low elevation) in the common garden experiment. Population, seed family and experimental block are considered as random effects in the model.

Source of variation	Fixed effects	Origin	Garden	O × G	Random effects	Population	Seed family	P × G	S × G	Block
** **	**d.f**	2,12	1, 8	2,12	** **					
**Germination probability**	**F**	2.27	0.01	1.15	**Var**	0.19	0.06	0	0	0.32
	**p**	0.150	0.940	0.350	**p**	0.030	0.140	-	-	0.096
Number of capitula*	**F**	0.42	648.05	1.42	**Var**	0	0	0	0	0.048
	**p**	0.669	<0.001	0.278	**p**	-	-	-	-	0.114
Plant biomass*	**F**	0.49	64.43	1.29	**Var**	0.0006	0	0	0.0267	0.041
	**p**	0.620	<0.001	0.310	**p**	0.430	-	-	0.0038	0.032
**Plant height**	**F**	2.24	211.34	0.18	**Var**	0.278	3.12	0	0	53.34
	**p**	0.150	<0.001	0.840	**p**	0.450	0.240	-	-	0.0287
Primary branch number*	**F**	1.00	67.13	0.13	**Var**	0	0.002	0.002	0.005	0.005
	**p**	0.400	<0.001	0.880	**p**	-	0.339	0.210	0.189	0.067
**Days to flower**	**F**	0.50	732.35	0.30	**Var**	0.000067	0.00168	0	0	0.0075
	**p**	0.619	<0.001	0.748	**p**	0.440	0.030	-	-	0.032
Specific leaf area*	**F**	0.26	0.00	1.78	**Var**	0.000016	0	0	0.0008	0.0065
	**p**	0.780	0.980	0.210	**p**	0.460	-	-	0.026	0.026
**Leaf dry matter content**	**F**	0.03	0.27	2.18	**Var**	0	0	0	0.0015	0.009
	**p**	0.970	0.610	0.160	**p**	-	-	-	0.005	0.025
**Mean leaf area**	**F**	1.70	25.48	0.69	**Var**	2.07	0	2.07	3.414	43.69
	**p**	0.220	0.001	0.520	**p**	0.250	-	0.250	0.175	0.027

F ratios and associated p values are given for fixed effects, variance estimates and p values of z tests are given for random effects.

The p values are not given for variance estimates set to zero.

*These variables were log transformed, Leaf dry matter content was logit transformed, and Days to flower was square root transformed.

#### Survival, growth and reproduction

**Fig 3:**
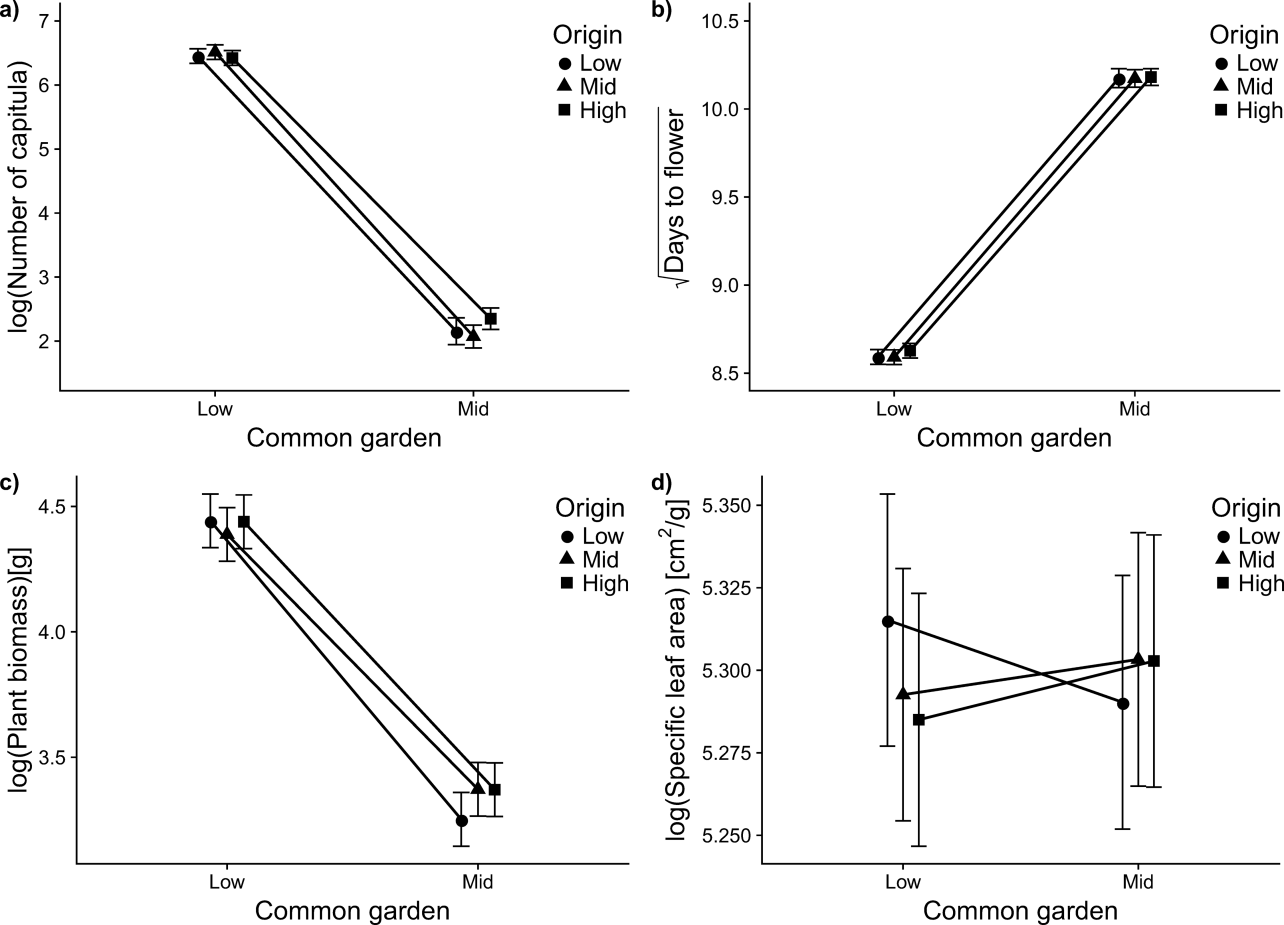
Lack of local adaptation in *Ageratina adenophora* populations. Reaction-norms of four important traits (a-d) of invasive *Ageratina adenophora* populations originating from three elevational belts (shown in the legend as Origin) in the western Himalayas to environmental conditions of the low (570m) and the mid-elevation (1330) gardens. Number of capitula, days to flower, and plant biomass differed significantly between gardens. None of them showed a significant origin effect or a significant garden × origin interaction (see Table 1 for results of statistical analyses). Plants belonging to low, mid and high origins are represented by circles, triangles, and squares respectively. The points show least square means and error bars represent standard errors obtained from the mixed effect models.

Survival of plants differed remarkably among the three gardens (F_2, 12_ = 73.78, p < 0.0001).While 99.5 ± 0.4% and 99.2 ± 0.5% of the plants survived at low elevation and mid elevation respectively, nearly all the plants died in the high elevation garden (0.5 ± 0.4%) most likely due to sub-zero temperatures. Owing to the extremely low numbers of surviving plants in the high elevation garden, we had to remove this factor level from subsequent analyses of traits.

(F_1,8_ = 221, p < 0.0001). Growth-related traits (i.e. biomass, plant height and a number of branches) differed significantly between the gardens suggesting large plasticity of these traits (Table 1, Fig 3). On average, the biomass of plants in the low-elevation garden was almost 3 times as large as the biomass of plants in the mid-altitudinal garden (Fig 3C Table 1). Furthermore, plants in the low-altitudinal garden were 1.8 times as tall and had 1.6 times as many branches as the plants from the mid-altitudinal garden. However, neither the origin of plants nor the origin × garden interaction had a significant effect, indicating an absence of population differentiation and local adaptation of these traits (Fig 3, Table 1). However, the seed family × garden interaction was significant for plant biomass suggesting intra-population genetic variation in the response of this trait to the environment.

We analysed reproductive fitness of individuals in two steps: first, the probability of flowering and second, the number of capitula (as a measure of reproductive output) of those individuals that produced flowers. While all surviving plants flowered in the low-altitudinal garden, only 18% of the surviving individuals flowered in the mid-altitudinal garden. The probability of flowering was low in the mid-elevation garden as merely 16.4 ± 2.0% of the surviving plants produced capitula compared to 97.2 ± 0.9% in the low-elevation garden. There was no significant effect of origin on flowering probability (F_2,12_ = 1.70, p = 0.22). The number of flower heads produced by those individuals that flowered (as measure of their reproductive output) was almost 50 times larger in the low-elevation garden (754.5 ± 44.6) compared to the mid-elevation garden (15.6 ± 60.7; F_2,12_ = 96.34, p < 0.0001), but there was neither a difference among the three origins (F_2,8_ = 0.05, p = 0.95) nor an origin × garden interaction (F_2,12_ = 0.06, p = 0.94) (Table 1).

**Fig 4:**
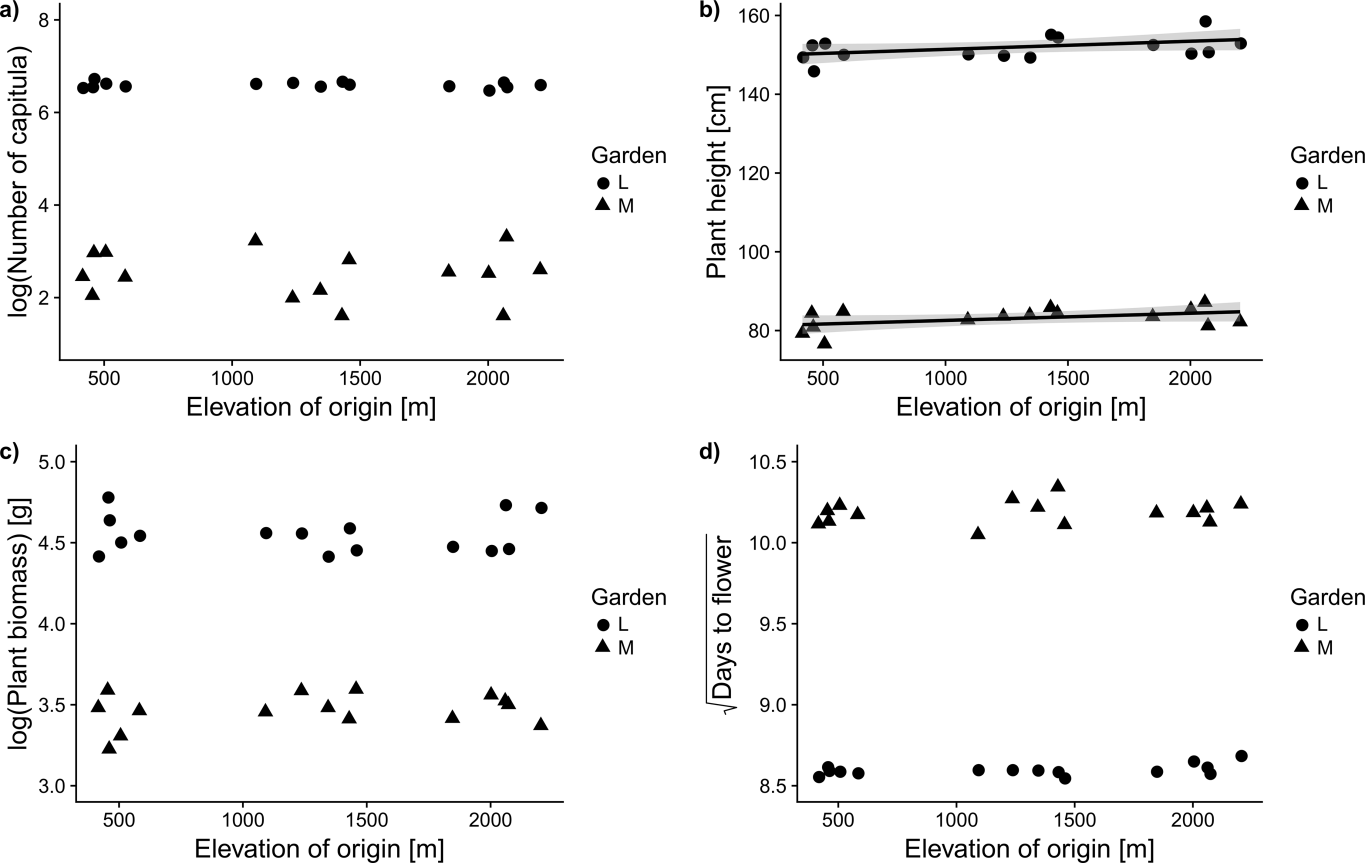
Absence of clinal differentiation of 15 invasive populations of *A*.*adenophora* along the elevational gradient for four important traits except for plant height (b) which shows significant but weak positive relationship. The scatter plots show the relationship between elevation at the home site of the 15 invasive *Ageratina adenophora* populations and their trait means measured in two common gardens (mid-elevation and low-elevation). Regression lines have been plotted for the only significant relationship, i.e. of plant height with elevation (F_1,26_ = 6.68, p = 0.0157, see Table 2 for details of ANCOVA results). Solid circles and triangles represent low and mid-elevation garden respectively.

Among all the nine growth- and fitness-related traits measured, only plant height showed a clinal variation along the altitudinal gradient: In both gardens, plant height increased significantly with the elevation from which the populations originated (Fig 4B, Table 2). Furthermore, the probability of flowering in the mid-elevation garden showed a marginally significant increase (F_1,12_ = 3.20, P = 0.097) with the elevation from which the populations originated (Table 2). We did not find any evidence for clinal variation along elevation in any other measured trait.

**Table 2:** Results of ANCOVAs to test for clinal differentiation among invasive *Ageratina adenophora* populations along the elevational gradient.

Source of variation		Elevation		Garden		Elevation × Garden	
Traits	df	F	P	F	p	F	p
Plant biomass*	1, 26	0.31	0.580	683.99	<0.001	0.41	0.527
**Plant height**	1, 26	6.68	0.016	5063.62	<0.001	0.026	0.874
Primary branch number*	1, 26	0.08	0.775	273.94	<0.001	0.06	0.809
Specific leaf area*	1, 26	0.24	0.627	0.22	<0.001	1.72	0.201
**Leaf dry matter content**	1, 26	0.53	0.820	6.16	<0.0199	3.17	0.868
**Leaf area**	1, 26	0.06	0.817	357.70	<0.001	0.87	0.358
**Days to flower**	1, 26	1.78	0.194	5717.07	<0.001	0.01	0.938
Number of capitula*	1,26	0.15	0.699	846.17	<0.001	0.05	0.820
**Probability of flowering**	1,13	3.20	0.097	-	-	-	-

Elevation of the home site of each population was used as a covariate, and garden as a factor. Please note that probability of flowering could only be tested for the low-elevation garden.

*These variables were log transformed, Leaf dry matter content was logit transformed, and Days to flower was square root transformed.

#### Leaf functional traits

Of the leaf traits measured, only mean leaf area showed a plastic response to the garden environments: in the mid-elevation garden, leaf area was reduced by almost one third compared to the low-elevation garden, but there was no garden × origin interaction (Fig 3, Table 1). Specific leaf area and leaf dry matter content did not show any significant effect of either garden, origin or their interaction. While we did not detect an origin × garden interaction for any of the leaf functional traits, we did find a significant seed family × garden interaction in the case of SLA and LDMC. Additionally, we found no evidence for clinal differentiation along the elevational gradient for any of the leaf functional traits (Table 2, Fig 4).

## Discussion

### Distribution pattern and life history stages

Our field survey revealed clear evidence for a unimodal distribution of invasive *A*. *adenophora* populations along the elevational gradient in the western Himalaya: The probability of occurrence peaks at 1320 m a.s.l., and steeply declines at both ends of the elevational gradient. Although we did not observe a complete absence of *A*. *adenophora* from lower elevations in our study area, the species has a distinct lower range limit and is not reported from the plains [54]. Physiological tolerance of a species to abiotic conditions plays a crucial role in determining the pattern of distribution along latitudinal or elevational gradients [62]. This should be particularly true for the upper range margin at high elevations [63]. Indeed, our common garden experiment suggests that the upper range limit is determined by low temperature (sub-zero) in winter, as plants failed to survive the winter at the uppermost site. Interestingly, during our field surveys, we observed that *A*. *adenophora* populations were confined mostly to habitats that are likely to provide shelter during winter such as steep rocky slopes and to forest sites.

In contrast to the upper range limit, the lower range margin is thought to be primarily shaped by biotic interactions [63]. However, our results suggest that even the lower range margin of a species may be predominantly determined by abiotic condition conditions if the environmental gradient is large enough as in the Himalayas. The distributional range of *A*. *adenophora* in western Himalaya spans a thermal gradient of roughly 11°C, which is much larger than most studies conducted in temperate regions. It has been proposed that a symmetrical and steep response is to be expected if abiotic stress controls the abundance pattern of the species while the response is likely to be skewed if biotic interactions play a crucial role [63]. The distributional pattern we observed is, however, largely symmetrical and steep, suggesting a crucial role of abiotic conditions also towards the lower end of the elevational gradient. Habitats of the lowermost populations in our study area are characterized by high summer temperature (around 37°C in the low-elevation garden, see S2 Table) coupled with dry spells. Under these conditions, seeds completely failed to germinate in our experiment. The optimal temperature for seed germination of *A*. *adenophora* is 25°C, and temperatures above 35°C are detrimental for germination [64]. High temperature coupled with desiccation may, therefore, have inhibited seeds from germinating in our experiment. Accordingly, our field survey indicated that *A*. *adenophora* populations in the lower elevational limit were specifically confined to ravines and water channels suggesting the requirement of higher soil moisture at the time of germination in summer.

Hence, our common garden experiment revealed that different life-history transitions are particularly vulnerable at the lower and the higher end of the elevational gradient, respectively: while winter survival appeared to be the most important determinant of the upper range limit, seed germination was crucial at the lower range margin. We, therefore, conclude that, although plants growing in the lowermost garden had the highest biomass and reproductive fitness, environmental conditions at mid-elevation are most favourable as they allow *A*. *adenophora* populations to successfully complete crucial life-stages, i.e. to germinate, survive, and reproduce.

### Absence of rapid evolutionary changes

To detect population differentiation and local adaptation of *A*. *adenophora* populations along the elevational gradient, we applied two approaches: First, we combined the common garden approach with reciprocal transplantation, and second, we searched for a relationship between various phenotypic traits measured in the common garden and the elevation from which the populations originated. However, our experiments revealed neither evidence for local adaptation nor any evidence for clinal variation among *A*. *adenophora* populations. Our result is in contrast to other case studies showing that adaptive divergence among populations may facilitate the range expansion of invasive plant species along environmental gradients. For instance, *Lythrum salicaria* [13] has been found to be locally adapted along a latitudinal gradient in North America, and *Solidago altissima* [43], as well as *Senecio inaequidens* [65], have shown clear-cut patterns of clinal differentiation along an environmental gradient in the invasive range. However, several studies have also reported the absence of genetic differentiation between invasive plant populations. For example, *Buddleja davidii*, [40] and *Mahonia aquifolium* [66] have spread across different habitats without showing evidence of local adaptation. In such cases, preadaptation to conditions in the invasive range [67] and high phenotypic plasticity [68,69] have been attributed to the success of invasive populations. For example in invasive *Acer negundo*, high amount of pre-adapted phenotypic plasticity plays a crucial role [70]. We included seed weight as a covariate to test any evidence of maternal effect manifested due to the provisioning of nutrition. However, there are several other mechanisms by which maternal environments can influence offspring (e.g. epigenetic changes). Although we cannot rule out that some of them might have influenced our experimental results, our data (as many other data from common garden experiments) do not allow estimating the magnitude of these effects. Nevertheless, we found almost no evidence for local adaptation in phenotypic traits despite huge differences among maternal environments.

In our study, there was an overall lack of strong evidence for clinal differentiation along an elevational gradient in most traits that we recorded. Several processes might have inhibited adaptive differentiation after the invasion western Himalaya. First, the plant has been reported to be a triploid with 51 chromosomes [71]. Triploid plants are not capable of undergoing the normal process of meiosis and hence they do not produce seeds by the sexual process [47]. Developmental studies of this species have further indicated that the embryo development may initiate even before meiosis and fertilization, which implies that the plant is capable of producing seeds apomictically [37,47,48,64]. Lack of sexual reproduction thus locks the existing genetic variation in the population by preventing recombination, and may, therefore, reduce its potential for adaptive evolution. Second, it is rather likely genotypes are “swapped” among populations and elevational belts due to seed dispersal by the wind, water or animals. We admit that the lack of gene flow by pollen among populations may then facilitate a pattern of population differentiation according to random drift or isolation by distance, in addition to any local adaptation that may have taken place. Third, herbarium records indicate that *A*. *adenophora* was introduced in 1927 to Western Himalaya as an ornamental plant in the experimental garden of the Forest Research Institute, Dehradun. If the current populations in Western Himalaya have originated from the initial introduction event, it is likely that the plant suffered from a genetic bottle neck. A period of 90 years since introduction may not be sufficient for the plant to accumulate enough genetic variation to undergo local adaptation, especially because of its apomictic nature. Although there is evidence (herbarium voucher number CNH-225216, central national herbarium, botanical survey of India) of separate introduction in events in the hills of peninsular India (botanical garden of Ootacamund, Tamil Naidu) prior to the introduction in Western Himalaya, chances that these geographically isolated populations have intermingled are rather scarce. Finally, the existence of a high degree of phenotypic plasticity of *A*. *adenophora* [72] suggests that plasticity may have contributed to invasive spread across a broad environmental gradient without undergoing adaptive evolution. Indeed, the large differences in trait values between our common gardens on the origin, population, and seed family levels suggest a high degree of phenotypic plasticity in this species.

Genotype × environment interactions signify differential response of genotypes to different environmental conditions. From an evolutionary perspective, genotype × environment interactions provide the basis of adaptive divergence in response to different environmental conditions. In our experiment, we detected significant Seed family × Garden interaction term for biomass, SLA and LDMC. This interaction indicates the existence of intra-population genetic variation in the response to the environment for various traits, which in turn may provide the opportunity for adaptive evolution in future. In addition, accumulation of mutations can further increase the genetic diversity of *A*. *adenophora* populations in the long run [26]. However, our results together with findings of other studies strongly suggest that recent invasive spread of *A*. *adenophora* is mostly due to its high phenotypic plasticity. Plastic responses across a broad range of environmental conditions may be crucial for the success of a triploid and apomictic species that lacks sexual recombination [73]. According to our results, further spread of *A*. *adenophora* to higher elevations is currently constrained by its sensitivity to low temperature.

## Supporting information

S1 Appendix About the plant. (DOCX)

S2 Appendix Survey map. (DOCX)

S1 Dataset Complete data set. (XLSX)

S1 Table Populations-Climatic variables. (DOCX)

S2 Table Common gardens-Climatic variables. (DOCX)

S3 Table Experimental dates. (DOCX)

S4 Table Common gardens-Nutrient concentration. (DOCX)

S5 Table Result of hierarchical regression models using ‘eHOF’ package in R. (DOCX)
