# Supplementary material for: Processes affecting altitudinal distribution of invasive Ageratina adenophora in western Himalaya: The role of local adaptation and the importance of different life-cycle stages
Source: PLoS One. 2017 Nov 10;12(11):e0187708. doi: 10.1371/journal.pone.0187708 (PMC5695283; doi:10.1371/journal.pone.0187708)
Supplement: S1 Appendix — (DOCX) [file pone.0187708.s001.docx]

**S1 Appendix. Study species**

*Ageratina adenophora* (Spreng.) King & H.Rob.

Syn: *Eupatorium adenophorum* Spreng.

It is an erect, branched perennial herb growing up to 1 m in height, flowering profusely in spring (March-April) with dense white terminal capitula borne in clusters. Each head bears 60-70 disc florets and a mature plant may produce as many as 2000 heads in the first year of growth. The plant keeps growing in the subsequent years by producing numerous branches from the base of the main plant, thus forming a dense, monospecific thicket. The branches produce adventitious roots when it comes in touch with moist soil in rainy season which further augments the lateral spread of the plant. The seeds are equipped with pappus facilitating air and animal dispersal. The plant shows luxuriant growth in cool moist regions along the slopes of hills or mountains but it is capable of growing in diverse conditions ranging from flat flood plains of lower Himalaya to steep and dry rocky slopes. The plants invade new regions along road verges and rivers which form a conduit for its dispersal^[[1]](#footnote-1)^.


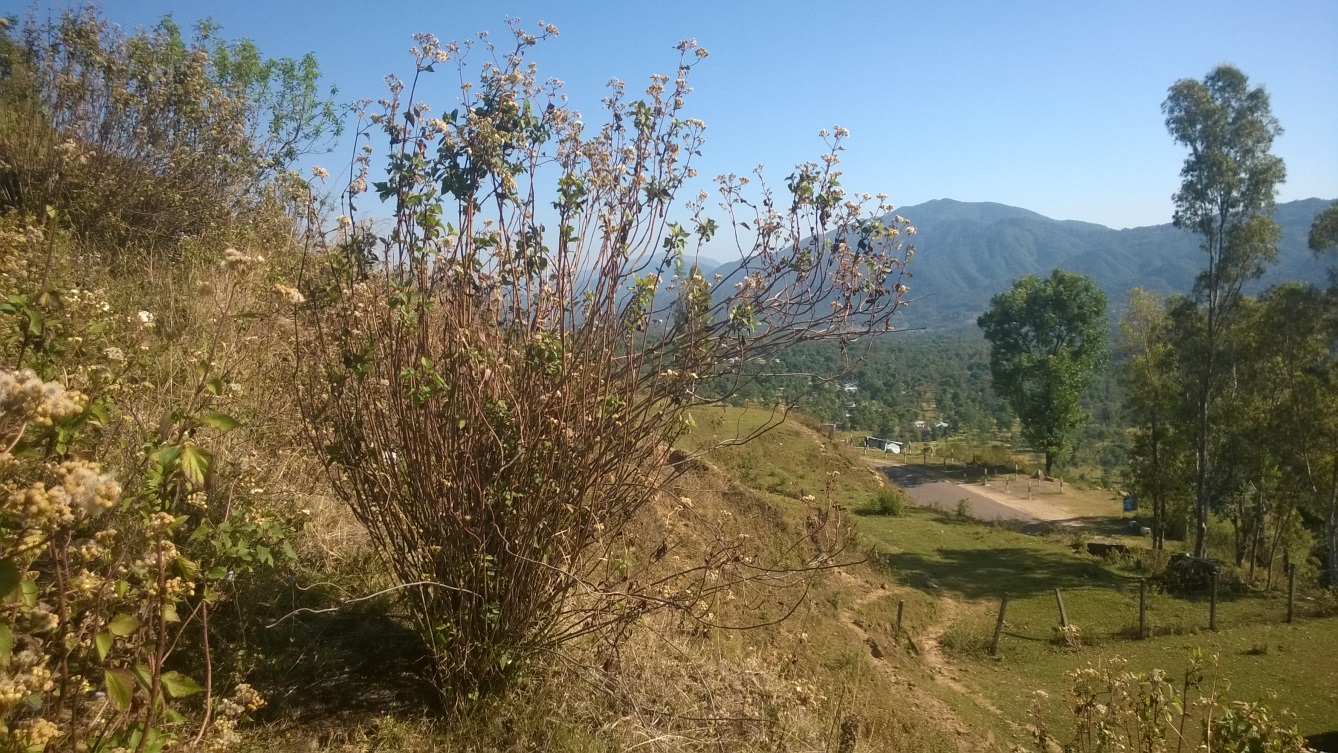


Invasion of *Ageratina adenophora* in Himachal Pradesh, India

1. Lu Z, Ma K. Spread of the exotic croftonweed (*Eupatorium adenophorum*) across southwest China along roads and streams. Weed Sci. 2006;54: 1068–1072. doi: 10.1614/WS-06-040R1.1 [↑](#footnote-ref-1)
