# Supplementary material for: Processes affecting altitudinal distribution of invasive Ageratina adenophora in western Himalaya: The role of local adaptation and the importance of different life-cycle stages
Source: PLoS One. 2017 Nov 10;12(11):e0187708. doi: 10.1371/journal.pone.0187708 (PMC5695283; doi:10.1371/journal.pone.0187708)
Supplement: S2 Appendix — (DOCX) [file pone.0187708.s002.docx]

**S2Appendix:**

Map showing the survey locations (n = 389) of *Ageratina adenophora* located in the western part of Himalayas. The presences (n = 193) and absences (n = 196) are depicted using red and blue coloured circles respectively. The elevation of the surveyed location in meters is indicated in by the number next to the circle.
