# Supplementary material for: Processes affecting altitudinal distribution of invasive Ageratina adenophora in western Himalaya: The role of local adaptation and the importance of different life-cycle stages
Source: PLoS One. 2017 Nov 10;12(11):e0187708. doi: 10.1371/journal.pone.0187708 (PMC5695283; doi:10.1371/journal.pone.0187708)
Supplement: S1 Table — (DOCX) [file pone.0187708.s004.docx]

**S1 Table.** Geographic coordinates, elevation and major climatic variables [1] of home sites of the 15 *Ageratina adenophora* populations used for the common garden and germination experiments. All the populations are located in Western Himalaya (Himachal Pradesh, India).

| Location | Latitude [°N] | Longitude [°E] | Elevation a.s.l. [m] | Mean annual temperature [°C] | Mean annual precipitation [mm] | Max temperature of Coldest Month [°C] | Min Temperature of Warmest Month [°C] |
| --- | --- | --- | --- | --- | --- | --- | --- |
| Balaru (Dehra) | 31.8288 | 76.2597 | 416 | 23.2 | 1523 | 38.7 | 6.7 |
| Jwali Bridge | 32.1468 | 76.0234 | 454 | 22.9 | 1854 | 38.9 | 6.4 |
| Jwali Stone Grinder | 32.17 | 76.031 | 460 | 22.7 | 1956 | 38.4 | 6.4 |
| Sandhol | 31.8864 | 76.6345 | 582 | 22.4 | 2155 | 37.1 | 6.9 |
| Sujanpur | 31.8383 | 76.5111 | 506 | 22.9 | 1986 | 38 | 6.9 |
| Baijnath | 32.0548 | 76.658 | 1091 | 20.2 | 2182 | 33.6 | 6.3 |
| Dhramshala | 32.2168 | 76.3347 | 1344 | 17.9 | 2612 | 30.4 | 4.7 |
| Jogindernagar | 32.0031 | 76.7711 | 1236 | 19.4 | 1920 | 32.3 | 5.7 |
| Palampur | 32.116 | 76.5653 | 1429 | 18.6 | 2422 | 31.2 | 5.4 |
| Rakh | 32.1493 | 76.4752 | 1457 | 18.3 | 2544 | 30.8 | 5.2 |
| Lower Birni | 32.1384 | 76.5614 | 2002 | 15.8 | 2254 | 27.9 | 2.7 |
| Billing | 32.0549 | 76.7396 | 2203 | 14.4 | 2057 | 26.3 | 1.2 |
| Jhitingiri | 31.948 | 76.8814 | 2059 | 15.5 | 1987 | 27.5 | 2.6 |
| Macleodganj | 32.2408 | 76.3306 | 1846 | 16.9 | 2475 | 29.2 | 3.7 |
| Jia Power Project | 32.1963 | 76.4852 | 2072 | 15.6 | 2274 | 27.7 | 2.4 |

1. Hijmans RJ, Cameron SE, Parra JL, Jones PG, Jarvis A. Very high resolution interpolated climate surfaces for global land areas. Int J Climatol. 2005;25: 1965–1978. doi: 10.1002/joc.1276
