# Supplementary material for: Processes affecting altitudinal distribution of invasive Ageratina adenophora in western Himalaya: The role of local adaptation and the importance of different life-cycle stages
Source: PLoS One. 2017 Nov 10;12(11):e0187708. doi: 10.1371/journal.pone.0187708 (PMC5695283; doi:10.1371/journal.pone.0187708)
Supplement: S2 Table — (DOCX) [file pone.0187708.s005.docx]

**S2 Table.** Geographic coordinates, elevation, and major climatic variables [1] of sites used for common garden and germination experiments in western Himalaya (Himachal Pradesh, India)

| **Common garden** | **Latitude [°N]** | **Longitude [°E]** | **Elevation a.s.l. [m]** | **Mean annual temperature [°C]** | **Mean annual precipitation [mm]** | **Mean maximum temperature [°C]** | **Mean minimum temperature [°C]** |
| --- | --- | --- | --- | --- | --- | --- | --- |
| **High-elevation garden** | 32.1424 | 76.5608 | 2098 | 14.7 | 2151 | 26.7 | 1.5 |
| **Mid-elevation garden** | 32.1065 | 76.5596 | 1331 | 18.9 | 2428 | 31.5 | 5.5 |
| **Low-elevation garden** | 31.8554 | 76.5028 | 572 | 22.5 | 2025 | 37.4 | 6.8 |

1. Hijmans RJ, Cameron SE, Parra JL, Jones PG, Jarvis A. Very high resolution interpolated climate surfaces for global land areas. Int J Climatol.; 2005;25: 1965–1978. doi: 10.1002/joc.1276
