# Supplementary material for: Processes affecting altitudinal distribution of invasive Ageratina adenophora in western Himalaya: The role of local adaptation and the importance of different life-cycle stages
Source: PLoS One. 2017 Nov 10;12(11):e0187708. doi: 10.1371/journal.pone.0187708 (PMC5695283; doi:10.1371/journal.pone.0187708)
Supplement: S3 Table — (DOCX) [file pone.0187708.s006.docx]

**S3 Table**: Table showing key experimental dates of common garden experiments. Please note that due to mortality of the plants at high elevation (>99%) biomass could not be harvested; similarly seeds did not germinate in the germination experiment at the lowermost garden.

| **Site** | **Sowing of seeds for main experiment** | **Transplantation of seedlings**  **for main experiment** | **Sowing of seeds for germination experiment** | **Counting of germinated seeds** | **Date of Biomass Harvest** |
| --- | --- | --- | --- | --- | --- |
| **High-elevation garden** | 3-7-2014 | 23-8-2014 | 27-7-2014 | 31-8-2014 | ------------ |
| **Mid-elevation garden** | 6-7-2014 | 16-8-2014 to  18-8-2014 | 23-8-2014 | 5-9-2014 | 15-8-2015 and 16-8-2015 |
| **Low-elevation garden** | 8-7-2014 | 18-8-2014 | 1-8-2014 | ------------ | 22-8-2015 and 23-8-2015 |
