# Supplementary material for: Processes affecting altitudinal distribution of invasive Ageratina adenophora in western Himalaya: The role of local adaptation and the importance of different life-cycle stages
Source: PLoS One. 2017 Nov 10;12(11):e0187708. doi: 10.1371/journal.pone.0187708 (PMC5695283; doi:10.1371/journal.pone.0187708)
Supplement: S4 Table — (DOCX) [file pone.0187708.s007.docx]

**S4 Table.** Concentration (mean ± sd) of important nutrients at the three experimental gardens (High, Mid, Low).

| **Soil parameters** | **High (n = 12)** | **Mid (n = 13)** | **Low (n = 14)** |
| --- | --- | --- | --- |
| **Ca [mg/kg]** | 1716 ± 91 | 2061 ± 699 | 1578 ± 82 |
| **K [mg/kg]** | 2366 ± 141 | 2331 ± 206 | 4931 ± 255 |
| **Mg [mg/kg]** | 1912 ± 263 | 2861 ± 378 | 7997 ± 344 |
| **P [mg/kg]** | 684 ± 46 | 400 ± 38 | 2445 ± 10 |
| **N [%]** | 0.26 ± 0.04 | 0.07 ± 0.01 | 0.09 ± 0.01 |
| **C [%]** | 2.89 ± 0.44 | 0.78 ± 0.10 | 1.10 ± 0.08 |
