# Supplementary material for: Processes affecting altitudinal distribution of invasive Ageratina adenophora in western Himalaya: The role of local adaptation and the importance of different life-cycle stages
Source: PLoS One. 2017 Nov 10;12(11):e0187708. doi: 10.1371/journal.pone.0187708 (PMC5695283; doi:10.1371/journal.pone.0187708)
Supplement: S5 Table — (DOCX) [file pone.0187708.s008.docx]

| **Model** | **Shape of the response curve** | **Deviance** | **logLikelihood** | **AICc** | **∆AIC** |
| --- | --- | --- | --- | --- | --- |
| I | Straight line-Null model | 539.2042 | 269.602 | 541.2146 | 22.6169 |
| II | Logistic - increasing trend | 538.4934 | 269.247 | 542.5245 | 23.9269 |
| III | Logistic - increasing trend but below the upper bound | 521.2176 | 260.609 | 527.28 | 8.6823 |
| ***IV*** | ***Unimodal symmetric pattern*** | ***512.5353*** | ***256.268*** | ***518.598*** | ***0*** |
| V | Unimodal skewed pattern | 512.5350 | 256.268 | 520.6392 | 2.0416 |
| VI | Bimodal pattern | 512.5353 | 256.268 | 520.6395 | 2.0418 |
| VII | Bimodal pattern | 512.5353 | 256.268 | 522.692 | 4.0943 |

**S5 Table.** Result of hierarchical regression models using eHOF package in R [1,2]. The probability of occurrence along elevational gradient was predicted using presence- absence data of *Ageratina adenophora.* Seven models with increasing level of complexity were fit using maximum likelihood procedure. Model IV had the lowest AIC value indicating unimodal and symmetric pattern of distribution of *Ageratina adenophora* along elevational gradient.

References:

1. Jansen F, Oksanen J. How to model species responses along ecological gradients - Huisman-Olff-Fresco models revisited. J Veg Sci. 2013;24: 1108–1117. doi:10.1111/jvs.12050

2. Huisman J, Olff H, Fresco LFM. A hierarchical set of models for species response analysis. J Veg Sci. 1993;4: 37–46.
